# Supplementary material for: Are carbon emissions trading and green financial instruments synergistic? -Comprehensive quantitative research based on content analysis
Source: PLoS One. 2024 Mar 7;19(3):e0298601. doi: 10.1371/journal.pone.0298601 (PMC10919601; doi:10.1371/journal.pone.0298601)
Supplement: S1 Data — (ZIP) [file pone.0298601.s001.zip › Data/地级市Z_F工作报告.zip!/║┌┴·╜¡/║╫╕┌╩╨/└┤╫╘╣1⁄2╓┌║┼í╛┬φ┐╦╩2╛▌═°í┐_2009.docx]

各位代表：

　　我代表市政府向大会作政府工作报告，请审议。请政协委员和其他列席的同志提出意见。

　　一、2008年主要工作回顾

　　过去的一年，我们在市委的领导下，坚持以科学发展为第一要务，以改善民生为根本目标，以安全稳定为第一责任，以自身建设为有效保证，积极克服各种困难，圆满完成了全年各项任务，形成了经济社会加快发展的新局面。

　　——经济运行态势良好，主要指标超额完成。地区生产总值预计完成184亿元，比上年增长12.2％，连续六年实现两位数增长；固定资产投资完成72亿元，增长30％；财政收入首次突破20亿元大关，实现22亿元，增长23.6％，其中一般预算收入完成9.3亿元，增长20％，比预期高7个百分点；城镇居民人均可支配收入达到10003元，增长10.2%，农民人均纯收入达到4618元，增长11.7%。工业增加值增长18.8％，利税增长21.5％；招商引资到位资金85亿元，增长30％；金融机构存贷款余额分别达到219.9亿元、142.2亿元，比年初增长25.6%、9.2%。

　　——项目建设实现重大突破，园区发展取得实质性进展。新一轮三年“项目建设年”开局顺利，实施500万元以上各类项目233个，完成投资47亿元。工业项目快速推进。实施重点工业项目42个，其中亿元以上项目9个，完成投资21亿元，增长133%。华鹤百万吨甲醇和新华百万吨化肥两大项目正式开工建设，成为我省东部煤电化基地建设的标志性项目。嘉晨120万吨洗煤、圣元580吨鲜奶等36个项目投产运行；兴汇100万吨玉米深加工二期、鹤矿集团10万千瓦矸石热电等6个项目正在建设；鹤矿集团120万吨鸟山煤矿项目完成国家审批手续，龙煤集团与禄恒集团合作的120万吨甲醇项目完成省级审批。对上争取项目资金取得实效。有37个项目列入国家支持计划，新华百万吨化肥、双兰星药业、小鹤立河水库消险扩建等项目获得第一批中央预算内投资补助2亿元，其中百万吨化肥成为东北三省争取中央支持资金最多的工业项目。工业园区建设全面推进。完成了园区整体规划，调整了煤化工产业发展规划，我市第一个“省级工业示范基地”获得省政府批准，结束了没有省级工业园区的历史。此外，为应对金融危机，及时制定实施了《支持中小企业发展的措施》。

　　——农业经济快速发展，新农村建设全面推进。农业生产实现历史突破。粮食生产喜获丰收，地方总产达13.65亿斤，区域总产达到59亿斤，均创历史最高水平；有机、绿色、无公害食品种植面积增加18万亩，绿标增加44个，“华池佐味”被评为我市第一个调味品绿标。畜牧业实现增加值2.8亿元，增长10％，生猪、奶牛、肉牛饲养量分别增长11％、9.7%和9.5％。农机农技水平明显提高。争取专项补贴1178万元，更新改造农机具近千台，农机化水平步入全省先进行列；绥滨县农业科技园区被评为全省县级样板。产业化经营扎实推进。泰丰粮油、兴汇粮食加工成为我市第一批国家级龙头企业，“梧桐”牌大米已申报“中国驰名商标”。农业基础设施建设加快。江萝灌区作为全省“千亿斤粮食产能工程”首批项目开工建设；投资4077万元，实施了小鹤立河、元宝山水库除险加固等8项重点水利工程；全省第一个市级黑土地水土流失治理工程完成投资500万元。新农村建设取得成效。首批28个省级试点村一次通过验收。投入607万元，解决了农村1.8万人饮水安全问题。改造泥草房2629户，整村改造14个，在全省率先完成年度任务，受到省政府表扬。

　　——第三产业逐步发展，整体活力不断增强。旅游业发展开创新局面。以“两峡一岛”界江游开通和黑龙江流域博物馆开馆为标志，旅游业进入新的发展阶段，接待游客增长78.4%，旅游收入增长9.4%。商贸流通业快速增长。完成增加值18.7亿元，增长17％，占全市经济的16％，实现税收1.58亿元，增长33.25％，占全市税收总额的9％。社会消费品零售总额实现51亿元，增长19.8％。对外贸易实现新突破。进出口额完成4400万美元，增长21.4％；利用外资1015万美元，增长65%，增幅列全省第六位。赴俄锰矿项目完成了融资和矿石取样，黄金项目进入详勘，磷矿项目已与俄方签订协议；首次在尼日利亚开发了锰、黄金等资源型项目，并实现了当年生产。

　　——75件大事实事按计划落实，各项事业全面进步。75件大事实事完成52件，其中18件超额完成；因上级政策调整，有2件延期实施；其余21件跨年度工程完成预定计划。此外，新增了“集体企业退休人员全部纳入基本养老保险、为参险企业退休人员发放御寒补贴、为城市低收入家庭发放临时生活救助金、解决第二阶段企业职工历史工资拖欠问题”等12项实事。启动了全市经济、国土、环保普查工作。积极支援四川抗震救灾，捐款捐物1554万元，派出援助人员22人。为完成各项大事实事，市财政直接增加投资2.07亿元。

　　民生事业实现新发展。新增就业2.5万人，发放小额担保贷款1000万元，“零就业”家庭动态管理为零，城镇登记失业率控制在3.9%以内。筹资2.04亿元，在全省率先解决了3.3万名国有和集体企业职工的历史工资拖欠问题，受到省政府表扬；机关事业单位人员调资和各项津贴补贴全部兑现；提高了城乡低保标准，超过全省平均水平；增加助学、扶贫、大病救助专项资金1500万元，对特殊弱势群体进行了救助和资助；分配廉租房205户，发放租房补贴8700多户。

城乡建设再上新水平。制定了《新一轮城乡建设重点工程五年规划》及《鹤岗市滨水城市规划建设方案》。350公里农村公路、12条城市主次干道投入使用；国土、矿山治理和供热三期工程、城市绿化、街巷路亮化、公园改造等任务全部完成；沉陷区改造完成年度计划，绥滨松花江大桥开工准备就绪。

　　社会事业取得新进展。教育事业持续发展，新建了特教中心食堂和宿舍，改善了5所学校校园，改造了9530平方米危房，办学条件进一步改善；高中阶段毛入学率达到78.25％，高考升学率达到86％，职业教育毕业生就业率达到90％，师专招生与就业工作取得新突破。卫生事业协调进步，新型农村合作医疗实现区域全覆盖，社区卫生服务人口覆盖率达到85％；突发公共卫生事件应对与救治取得良好成效，结核病、艾滋病防治水平走在全省前列；工农区被评为我市第一个省级卫生先进区。人口与计划生育考核指标超额完成。文化事业繁荣活跃，基础建设得到加强，铺设有线电视光缆74.5公里,新增用户5000户，实现区域电视新闻节目互转互播；广播电视节目在全省评比中获奖40个，文艺作品获省级以上奖励134项，均创历史最好水平。体育事业成绩喜人，2万平方米全民健身中心落成，5个市级训练基地建成；竞技体育获省级以上金牌37枚，夺得世界青年速滑锦标赛速滑项目1金1银，取得我国参赛最好成绩。科技工作逐步加强，申报国家、省级科技项目23项，引进海外技术14项，组建5户企业研发机构，开展了海外学人鹤岗行、农民科技节等活动。环境保护取得实效，制定了《加强节能减排实施意见》等10个文件，开工建设了我市第一个污水处理厂，实施了16个水污染减排项目，关停了8家小造纸厂，推进了电机节能和锅炉改造等13项节能工程。化学需氧量静削减2200吨，二氧化硫静削减200吨，单位生产总值能耗下降5%。

　　——加大工作力度，安全稳定基础工作得到强化。以专项整治活动和“百日安全行动”为契机，重点实施了标准化和本质安全型煤矿创建工作，加大了对人员聚集场所的排查力度，整改各类隐患3100处，关停不合格公共娱乐场所102家，查扣各类违法违章车辆531台。非煤工矿商贸、道路交通死亡人数分别下降37.5%、45.7%；连续13年未发生重特大火灾，连续3年未发生火灾亡人事故。加强公安“三基”建设，刑事案件立案数下降4.7％，破案数提高67.5％，其中命案起数下降42.6％，破案率达到93.5%。信访工作加大投入力度，解决了57件疑难积案，进京访下降13％，到省非正常集体访、个体访分别下降20％、74％。投入450万元，巩固国防边防，军地“五联”创建在全省介绍经验。

　　此外，政府自身建设和人事、民族、宗教、妇儿、外事、侨务、审计、监察、物价、双拥、人防、档案、司法、统计、地震、残联、老龄等事业扎实推进，取得新的成绩。

 在总结成绩的同时，我们也清醒地看到，按照科学发展观和人民群众的要求，与发展较快的市地相比，我市还存在许多差距和问题。特别是去年3月和9月，先后发生了两起重特大煤矿事故，死亡44人，造成了重大损失，带来了不良影响。在此，我代表市政府作出深刻检查。11月，我市又发生了“民间借贷和集资”引发的一系列问题，给部分群众和企业乃至全市的稳定发展造成了严重困扰，尽管做了大量艰苦细致的工作，但问题尚未得到解决。对此，市政府高度重视，将竭尽全力，妥善处理，给各方面一个高度负责的交待。

　　总之，2008年喜事多、难事多、大事多，是极不寻常的一年，我们经受住了来自经济、社会、安全、稳定等方面的重大考验，取得了来之不易的成绩。这是省委、省政府和市委正确领导的结果，是各级人大、政协有效监督和大力支持的结果，是各级组织、广大干部群众和中省直各单位、区域各单元共同奋斗的结果。在此，我代表市政府，向全市各族人民、各民主党派、各人民团体、各界人士和各方朋友表示衷心的感谢并致以崇高的敬意！

　　二、当前的总体形势

 正确判断形势，准确把握市情，特别是清醒认识面临的挑战与机遇，是做好一切工作的前提。

 当前，世界金融危机已从虚拟经济扩展到实体经济，从发达国家传导到新兴市场国家乃至我市这样一个偏远的欠发达城市。这种不利影响与我市的固有矛盾交织在一起，使加快发展面临着前所未有的挑战。突出表现在：

 一是对企业生存发展的挑战。由于煤炭价格下降，用电需求减少，致使鹤矿集团减收，华能鹤电公司亏损；地方工业和房地产、进出口等中小企业由于抵御风险能力弱，加之市场萎缩、价格下降、资金紧张，效益大幅下滑，亏损面达到25％。

　　二是对产业结构调整的挑战。我市产业结构不够合理，三次产业比例约为24：44：32，一产、三产较弱，且短期内升级和增长的空间较小。二产缺乏新的增长点，特别是煤炭业占工业的85％以上，提供的税收占财政收入的65％，且煤炭仍处于粗加工阶段，资源利用效益不高，产业结构调整、优化、升级将成为重要而紧迫的课题。

　　三是对保持较快增长的挑战。从投资看，危机对投资预期产生较大影响，加之国家产业政策调整，项目准入门槛提高，市场变化较大，工业项目建设形势不容乐观。从消费看，我市地处偏远，消费条件有限，群众平均购买力偏低，危机又对消费预期产生负面影响，短期内依靠消费拉动增长十分困难。从出口看，我市对外贸易只占经济总量的0.2％，出口加工企业仅有13家，且规模不大，加之受俄方有效需求不足影响，进出口贸易将减少。

　　四是对社会大局稳定的挑战。由于经济社会转型期的各种矛盾与危机的冲击相叠加，全市地方煤矿被迫停产与“民间借贷和集资”引发的一系列问题相交织，等等，使各种不稳定、不确定因素大量增加，就业、信访、治安等问题将更加突出。

　　五是科技人才力量不足的挑战。我市人才总量不足，结构和分布不合理，大都集中在煤炭、医疗、教育系统，而且长期面临“留人难、引人更难”的问题。我市科技力量薄弱，科技进步贡献率只有25％左右，对经济发展的先导、引领和支撑作用明显不强。这种现状，将使经济社会发展受到严重制约。

　　六是对财政正常运转的挑战。因企业效益下滑、增值税转型等政策调整，将使税收减少1.8亿元以上，且新增财源不足。同时，基础建设匹配、民生事业投入、社会事业发展、偿还历史欠账、工资津贴兑现等刚性支出大量增加，财政运转将更加艰难。

　　与此同时，我们还必须看到，鹤岗的发展更面临着前所未有的机遇。主要体现在：

　　（一）国家扩大内需政策措施的机遇。为应对危机，国家加快宏观政策调整，货币政策从适度从紧到适度宽松，财政政策从稳健到积极，随之出台扩大内需、改善民生、加强基础设施建设、金融业支持地方经济发展等一系列重大措施，必将给我市全面发展创造有利的大环境，为“保增长、保民生、保稳定”带来最有效的支持。

 （二）全省实施新一轮发展战略的机遇。我省确定了建设八大区域发展战略和十项重大工程，多数与我市有直接关系，必将对我市加快发展产生重大的拉动作用。“东部煤电化基地建设区”战略，为我市抢先建成现代煤电化基地创造了有利条件；“东北亚经济贸易开发区”战略，为我市实现对俄经贸战略升级创造了有利条件；“北国风光特色旅游开发区”战略，为我市把旅游业培育成新的经济增长点创造了有利条件；“千亿斤粮食产能工程”战略，为我市率先建成三江平原食品工业基地提供了有力条件；“现代交通网络建设工程”战略，为我市加快建成一批公路、铁路、港口项目提供了有力条件，等等。

　　（三）我市充分发挥自身优势的机遇。我市具有相对丰富的煤炭、木材、粮食、土地、水利、非金属矿产等资源。煤炭价格虽然暂时下滑，但作为“第一能源”的地位没有改变，为煤炭深加工特别是煤电化产业发展提供了条件；我市有充足的电力能源和劳动力等生产要素优势，为建设资源加工型、能源保障型、劳动密集型项目提供了有利条件；我市生态优势明显，土壤条件好，人均耕地多，食品加工基地基本形成，又有农垦的示范带动作用，为发展现代农业提供了有利条件。这一切，无论是应对危机还是迎接新一轮大发展，都是不可替代的宝贵优势。同时，我市加快发展的方向正确、思路对头，特别是经过近几年的艰苦努力，全市的项目建设、城乡建设、社会建设具备了较好的基础，加之全市人民有着求发展、盼发展、支持发展的强烈热情，都将为实现更快、更大、更好的发展创造有利的条件。

	总之，今年将是我市发展史上极为困难的一年，也是大有作为的一年。我们面临的形势虽复杂而严峻，但机遇大于挑战，动力大于压力。因此，我们必须保持清醒头脑，坚定发展信心，把“保增长、保民生、保安全、保稳定”作为主要任务，扎扎实实地办好自己的事情，抓好今年的工作，从而实现顺势而进、乘势而上。

　　三、2009年重点工作安排

　　今年是新中国成立60周年，是经受金融危机严峻考验的一年，也是深入学习实践科学发展观的关键一年。

 按照市委的战略部署，今年政府工作的总体要求是：深入贯彻落实科学发展观，把保增长作为首要任务，积极扩大内需，实现投资、消费、出口同时拉动，加快发展方式转变和产业结构调整；把保民生作为根本目标，努力提高人民群众收入水平，不断改善生产生活条件，完善社会保障体系；把保安全稳定作为重要责任，强化安全发展、和谐发展理念，全面加强安全生产和社会治安综合治理，妥善解决群众关注的热点难点问题。切实加强政府自身建设，积极应对挑战，抢抓发展机遇，努力争取更快、更大、更好发展，为实现“城市创新转型、人民富裕幸福”的目标奠定更加坚实的基础。

　　主要预期目标是：地区生产总值增长12%，财政收入增长12%，固定资产投资力争增长35%，社会消费品零售总额增长15%。工业增加值增长15%，利税增长15%；进出口额增长15％，利用外资增长10%。城镇居民人均可支配收入、农民人均纯收入分别增长8%、7%，城镇登记失业率控制在4.5％以内。二氧化硫排放量降低1.4%，化学需氧量降低8.5%，单位生产总值能耗下降7.5％。

　　为此，要重点抓好六个方面工作，实现三次产业协调发展、十项工程全面推进、两大保障坚强有力的目标。

　　（一）突出项目建设，大力发展新型工业

 坚持“工业立市”战略，强力推进“项目建设年”活动，以大项目为牵动，壮大优势产业，培育新兴产业，努力实现速度、效益、结构协调发展，逐步走上科技含量高、经济效益好、资源消耗低、环境污染少的新型工业化之路。

 加大工业项目建设力度。强力实施领导专项推进和激励约束机制，形成党委、人大、政府、政协四大班子抓项目，市、县区、乡镇、村四级组织上项目，全市人民参与招商引资的局面。实施千万元以上重点项目36个。要强力推进百万吨甲醇和百万吨化肥两个重大项目；加快焦化项目集聚发展，争取鹤翔90万吨焦化建成投产，鲲鹏、万昌、建龙三个焦化项目力争开工一个。加快风力发电项目建设，鹤林和三江风电项目完成测风，萝北风电项目力争开工建设，实现风能利用零的突破。积极发展高新技术项目，投资4500万元的建森五味子加工、投资1000万元的东新豆制品项目完成基础建设，中国建材研究总院授权的中岩新型建材项目和神华节能墙体材料二期工程投产。鹤矿集团矸石热电一期工程10万千瓦竣工投产，二期、三期工程做好前期工作；120万吨鸟山煤矿恢复建设；投资1690万美元的石墨深加工项目实现达产达效；秸秆发电、稻米深加工、酱业搬迁三个农垦项目全面开工；加快100万吨玉米深加工二期工程、万禾园油脂二期、双兰星药业、人和米业稻壳发电、禾友农药搬迁改造等项目建设，力争早日投产；100万吨磷肥、电厂三期、绥滨秸杆发电等项目完成招商、选址等前期准备。设立向上争取项目专项资金，全面加强项目前期工作，力争更多项目得到国家支持。

 加大工业园区建设力度。按照“统一规划、分步实施、重点突破、滚动发展”的原则，建设完善市级煤电化A区、B区、医药、食品、石墨五个分园区，力争实现各县区都有特色园区，各乡村都有特色项目。多元筹措资金，加强水、电、路、桥等基础设施建设；完善管理服务机制，出台优惠政策，重点引进科技含量高、见效快、税收就业贡献大的项目，培育新的经济增长点。

 加大对企业发展的支持力度。认真落实各项政策措施，突出解决资金“瓶颈”问题，用好1000万元中小企业发展专项资金；两年内再向财政担保中心注入4000万元资本金，加强银企对接，积极争取信贷支持。高度重视重点产业、重点企业发展，积极支持域内大企业应对危机。切实抓好地方煤炭企业发展，正确处理安全与生产的关系，加快对符合安全生产条件煤矿的复产验收，争取尽快开工生产，稳定煤炭产量；鼓励煤矿资源整合，把整合作为减少小煤矿数量的主要手段，合理安排关井计划，建立科学的退出机制，努力完成国务院、省政府下达的关井任务。大力支持老企业技术改造，引进高新技术和新项目，加强与发达地区产业转移的对接，加快产品结构调整，提高市场竞争力。出台具体政策，帮助房地产企业解决销售和动迁难题；坚持内企外企享受同等政策，所有工程项目在履行招投标程序的基础上优先本市企业。建立经济运行分析制度，强化领导包企责任制，帮助企业解决困难。

　　加大区域共建力度。充分发挥鹤矿集团、宝泉岭农垦分局、鹤北林业局、华能鹤电公司等大企业的人才、技术、装备和管理优势，全力支持域内重点项目、重大工程建设，积极推进市局场县在城市改造、原料基地建设、基础设施完善等方面共建共享。进一步支持县区发展，扩大县区经济总量，进一步形成合力上项目、携手抓发展的新局面。

　　（二）突出改革创新，大力发展现代农业

	全面落实十七届三中全会精神，以农业增产、农民增收、农村繁荣为重点，全面加强新农村建设，切实加快农村发展。

	积极推进农村改革深入化。加快农村体制机制创新步伐，积极稳妥地推进农民土地承包经营权流转，扩大规模经营，提高土地集约化水平；发展专业合作组织，提高农民组织化程度；加快农村金融改革，开展土地承包经营权抵押贷款业务，推动金融机构开办大型农机按揭贷款和融资租赁业务，逐步建立村镇银行。积极推进林权、户籍等制度改革。

积极推进农业生产基地化。要把食品工业作为战略产业来抓，把农业生产作为原料基地加快发展，壮大粮食、畜产品和绿特色食品加工业三大主业。围绕全省“千亿斤粮食产能工程”，按照高产、优质、高效的要求，调整玉米、水稻、大豆种植比例，高产高效作物增加11万亩，有机、绿色、无公害作物种植面积达到120万亩，粮食单产水平提高5%。围绕省“千万吨奶战略工程”、“五千万头生猪规模化养殖战略工程”，加快发展质量效益型畜牧水产业，抓好生猪、奶牛、肉牛基地建设，存栏力争增长7%以上。科学合理地安排木材生产计划，推进林下种植业、特色养殖业发展，培育龙头企业，打造林工产品精深加工基地。

　　积极推进经营方式产业化。加快绿色食品加工基地建设，扶持龙头企业发展。发挥三江、鹤鸣等米业集团优势，引导企业重组加入，增强集团实力，建成国家和省级水稻加工基地；加快壮大兴汇粮食加工、九三油脂、圣元和完达山乳业、北大荒肉业、双鹤食品等企业，牵动米、豆、乳、肉、禽等深加工产业健康发展。完善产业化利益联结机制，在股份制、股份合作制等方面迈出实质性步伐。

　　积极推进技术装备现代化。依托省农科院、东北农业大学等科研院所，建设市级“黑龙江流域现代农业科技示范园区”，两县各建成1个高标准现代农业科技示范园区，争取每个乡镇建设1个农业科技示范基地，每村建设1块科技示范田。加快农机化进程，争取资金4000万元，使农机规模经营达到20万亩以上。

　　（三）突出现代服务业，大力发展第三产业

　第三产业特别是现代服务业是衡量现代城市经济社会发展水平的重要标志，也是改善生产生活条件、增加就业和税收的重要保证。要抓住国家扩大内需、拉动消费的机遇，制定第三产业发展规划，全面发展生产性、消费性、公共性、基础性服务业，促进产业结构优化升级。

 努力提升旅游业发展水平。发挥我市界江、森林、石林、湿地、冰雪、人文、矿山、农场等旅游资源丰富的优势，突出生态特色，完善规划布局，抓好精品线路，全力打造“界江游”品牌。深度开发风光游、边境游、生态游、养生度假游等旅游产品，加大宣传力度，完成名山岛、细鳞河民俗风情园、太平沟“四A”级景区申报评定工作，努力建成优秀旅游城市，把旅游业培育成新的经济增长点。

 努力提升商贸物流业态发展水平。加快推进大商集团新玛特商场、台湾大润发集团大福源超市、红星——美凯龙建材家具广场等项目建设进度；修建工农、向阳露天集贸市场；引资1亿元，建成2个集销售、维修、配件和信息服务为一体的汽车商店。实施“商务惠民”和“万村千乡”市场工程，发展“满天星”式社区商服业，扩大农村连锁店覆盖面，推动“家电下乡”工程。开发服务性消费，提升餐饮、住宿、健身、文化、娱乐等传统服务业发展水平；推动休闲产业发展，培育“早餐经济”、“夜间经济”、“假日经济”等新的消费热点，引导健康文明的消费和生活方式。积极发展现代物流业，加快公路货运、新南粮食等物流中心建设，做大做强双鹤、泰丰农产品等4个物流中心和比优特超市连锁业，完善2个铁路战略装车点，规划进口锰铁和木材加工园区，发展第三方物流等新型业态。

努力提升对外经贸合作水平。抓住国家沿边开放战略和俄远东开发计划对接的机遇，加快向俄腹地延伸，扩大与其他国家的合作。设立50万元外贸发展专项资金，扶持外贸企业扩大对外经贸合作，增加机电、玻璃、建材、农药、木制品等产品出口。抓好赴俄锰矿开发采矿基地建设、石油天然气开采项目竞标、俄方氟化石墨和煤炭液化技术引进工作。

 努力提升金融业发展水平。支持金融机构拓宽服务领域，建立小企业金融服务专营机构，实现信贷快速增长。加强金融创新与监管，规范金融中介服务，积极启动民间资本，抓好小额贷款公司、企业贷款互助组织等试点工作。

　　（四）重点实施十项工程，努力办好百件实事

　　工程是社会进步的载体，也是全面系统谋划发展的科学方法；实事是政府履职的基点，也是人民群众利益的直接体现。要利用国家的政策机遇，在抓工程、办实事上进一步加大力度。今年重点实施十项工程，办好百件实事。

　　一是抓好文明城市建设工程。开展文明城市创建活动，坚持高标准规划、高质量建设、高水平管理，推进新一轮城乡建设工程；出台城市经营管理办法、动迁管理办法，加快推进“三供两治四化”工程，改善生活条件，强化承载功能，提高城市品位，努力把鹤岗建设成宜居、宜业、宜游、宜发展的城市。具体要办好25件实事。

　　1.投入2.4亿元，开工建设总投资8.5亿元的绥滨松花江公路大桥。

 2.争取开工建设总投资7000万元的鹤岗至萝北二级公路整修工程。

 3.全面启动总投资6.1亿元的鹤名一级公路前期工作，力争开工建设。

 4.投入6885万元，建设绥嘉公路兴东至太平沟段。

 5.投入2000万元，建设太平沟至嘉荫河大桥40公里公路。

 6.投入3500万元，全部完成通村公路建设任务。

 7.争取开工建设总投资5.6亿元的鹤名铁路。

 8.投资1000万元，修建10条城市道路。

 9.投入1400万元，改造4公里老国防路。

 10.投资360万元，修建3万平方米街巷路。

 11.投资240万元，安装街巷道路路灯。

 12.投资600万元，扩大城市绿化规模。

 13.投资300万元，美化南城门周边环境，建成百鹤园主题广场。

 14.投入3.5亿元，完成110千伏棚户区变电所、西郊变电所和220千伏鹤南输变电工程。

 15.投入1.67亿元，完成居民燃气储存、输送工程。

 16.投入460万元，完成1800米黑龙江名山段护岸工程。

 17.投资200万元，推进小鹤立河护岸工程。

 18.投入5100万元，改扩建热力公司供热设施。

 19.投入3000万元，建成南山区5000米供水管线。

 20.投入2200万元，迁建市第二看守所。

 21.投入430万元，在兴安区建设消防四中队。

 22.投资200万元，建成市应急处置中心。

 23.投资200万元，建设人民信访接待中心。

 24.投资400万元，启动建设公共资源交易中心。

 25.投入200万元，在工农区建成一个省级标准化社区。

　　二是抓好农业基础设施工程。改善农业生产条件，加强农田水利、防疫灭病、农情预警、畜牧养殖、农机装备等设施建设，增强抗灾减灾能力，提高农业综合效益。具体要办好18件实事。

　　1.投入1.1亿元，建设小鹤立河水库消险扩建大坝主体及溢洪道工程。

 2.投入1.65亿元，建设江萝灌区渠首、引渠、泵站。

 3.投入1180万元，建设元宝山水库除险加固工程。

 4.投入380万元，建设十里河水库除险加固工程。

 5.投入1000万元，修建农田水利设施，新增水田灌溉面积5万亩。

 6.投入440万元，建设20处农村饮水安全工程。

 7.投入1000万元，建设水稻育秧大棚1000栋、小区20个。

 8.投入460万元，建设生猪、奶牛标准化规模养殖场和养殖小区。

 9.融资3000万元，建设3个现代农机专业合作社。

 10.投入1000万元，加大农机直补力度。

 11.投入870万元，推进东方红乡土地整理项目，新增耕地40公顷。

 12.投入1000万元，实施农村沼气利用工程。

 13.投入400万元，建设林业局富森畜产公司沼气利用工程。

 14.融资1000万元，争取开工建设台湾农民创业园。

 15.投入5000万元，建设黑龙江流域现代农业科技示范园区。

 16.投入950万元，建设市动物疫病预防与控制中心。

 17.投入800万元，建设市马铃薯脱毒种薯快繁基地。

 18.投入300万元，建设省级新农村试点村15个。

　　三是抓好旅游综合开发工程。加强旅游资源开发和基础设施建设，提高景区可进入性，增强服务接待能力。具体要办好10件实事。

　　1.投入4000万元，建成萝北四星级宾馆。

 2.投入2000万元，建成黑龙江流域博物馆。

 3.投入600万元，建设名山岛民俗风情园。

 4.投资600万元，建设3000平方米太平沟旅游接待中心。

 5.投入100万元，完善桶子沟植物园原始森林景区。

 6.投入400万元，建设绥滨月牙湖生态景区。

 7.投入500万元，启动建设萝北水莲湿地公园。

 8.投入550万元，建设嘟噜河湿地保护公园，加强植被恢复和资源保护。

 9.投入500万元，建设金顶山龙掌岩石峰风景区。

 10.投入150万元，完善细鳞河民俗风情园。

　　四是抓好生态环境保护工程。以环境整治和发展循环经济为重点，全面贯彻落实《加强节能减排实施意见》和《单位GDP能耗统计监测及考核实施方案》，成立节能减排专门机构，设立专项基金和奖励资金，努力完成节能减排任务，加快生态城市建设。具体要办好15件实事。

　　1.启动总投资3亿元的鹤矿集团水泥厂搬迁工作。

 2.投入2.4亿元，完成华能鹤电公司3台机组脱硫改造。

 3.融资9700万元，建成城市生活垃圾无害化处理厂。

 4.融资1.8亿元，完成城市西部污水处理及再生水利用工程，启动建设东部污水处理工程。

 5.投入1200万元，实施鹤矿集团富力煤矿矿井水净化利用工程。

 6.投入3000万元，完成50万平方米既有建筑供热计量及节能改造。

 7.投入1500万元，继续建设矿山公园，力争“十一”开园。

 8.投入450万元，完成4万亩黑土地水土流失治理任务。

 9.投入370万元，综合治理森林重点火险区。

 10.全部完成五号水库水源地鹤林林场房屋搬迁任务。

 11.投入1500万元，实施10万亩封山育林、4万亩造林绿化工程，完成“三年绿化龙江大地”年度计划。

 12.投入560万元，建设细鳞河自然保护区。

 13.投入720万元，建设绥滨李家岛珍稀物种保护区。

 14.投入600万元，建设萝北乌苏里白鲑国家级资源保护区。

 15.投入360万元，建设萝北黑龙江鲟鳇鱼自然保护区。

　　五是抓好教育公平发展工程。坚持教育优先发展，深化教育教学改革，优化整合教育资源，积极改善办学条件，提高各级各类教育综合发展水平；促进教育公平，落实农村“两免一补”和城市义务教育免除杂费政策，推动城乡义务教育均衡发展，努力实现“学有所教”。具体要办好7件实事。

　　1.投资560 万元，扩建4800平方米教学楼，改造1000平方米学校危房。

 2.投入300万元，建设中小学生校外活动中心。

 3.投资200万元，维修改造部分学校锅炉。

 4.投资150万元，改善3所学校校园场地。

 5.投入300万元，资助7000名贫困学生。

 6.投入100万元，建成市一中过街天桥。

 7.投资60万元，创建5所省级标准化学校。

　　六是抓好医疗卫生服务工程。加快城市卫生服务网络建设，打造大医院与社区卫生机构共同发展的格局；深化农村医疗卫生改革，完善农村卫生服务体系；加强应急机制与体系管理建设，提高突发公共卫生事件处置能力；提升医疗卫生管理水平，全面落实计划生育惠民政策；开展“百千万”惠民义诊活动，努力实现“病有所医”。具体要办好7件实事。

　　1.投入1800万元,建设3所社区卫生服务机构。

 2.投入1400万元，建设2所二级综合医院。

 3.投资220万元,实施“婴幼儿健康促进计划”和计划生育家庭奖扶制度。

 4.投资35万元，建设2所中小学校人口素质教育示范基地。

 5.减免符合条件的残疾军人、军烈属和在乡复员军人医疗费。

 6.每区各确定一所惠民医疗机构，使大病救助对象就近得到医疗服务。

 7.投入206万元，开展“妇女健康促进工程”，免费为万名妇女进行健康普查。

　　七是抓好文化体育繁荣工程。实施文化精品工程，打造名牌节目，树立鹤岗新形象；整合文化信息资源，加强文化服务体系建设；深入开展全民健身运动，提高竞技体育实力，全面备战省第十二届运动会；加快文化、体育产业化、社会化进程，增强自我发展能力。具体要办好9件实事。

　　1.投资200万元，规划建设天水湖健身休闲广场。

 2.投入2000万元，启动有线电视数字平移改造。

 3.投入400万元，开通地面数字电视。

 4.投入200万元，解决电视连续剧《解放区的天》拍摄的前期费用。

 5.投资1400万元，推进市博物馆建设。

 6.投资600万元，建设市文化活动中心。

 7.投入240万元，新增全民健身路径30套。

 8.投入260万元，建设20个社区图书室和40个农家书屋。

 9.投资100万元，购置放映设备，为城乡群众免费放映500场数字电影。

　　八是抓好人才科技强市工程。充分发挥科技的先导作用和人才的支撑作用，加快对外科技合作，提高科技进步贡献率；加快人才队伍建设，提升人才综合素质，加强优秀拔尖人才评选奖励工作。具体要办好7件实事。

　　1.投入350万元，与省内高校联合规划建设“高层煤化工人才培训基地”。

 2.加强实用专业建设，培养100名煤化工技术人才、150名俄语和旅游应用人才，开展职业教育培训3万人次以上。

 3.建设师专与哈尔滨铁道技术学院联合职业培训基地，培养技能型人才。

 4.设立60万元专项资金，加强干部教育培训工作。

 5.投入100万元，建设20个农村科普大院。

 6.投入500万元，完成煤矿防灾三维地理信息系统硬件设施安装。

 7.与大专院校和科研单位联合组建5个企业研发机构。

　　九是抓好保障性安居工程。以解决中低收入家庭住房为重点，加快“一区两棚两房”改造和建设步伐，努力实现“住有所居”。具体要办好7件实事。

　　1.投入5.35亿元，启动兴安区棚户区改造工程，建设东山区沿河北小区、南山区平安小区，完善兴山区休闲小区。

 2.投入6500万元，争取完成兴山、向阳地区一氧化碳渗出区居民的整体搬迁工作。

 3.投入1.6亿元，改造农村泥草房2600户。

 4.投入2亿元，全部完成沉陷区治理工程。

 5.投入500万元，实施林区棚户区改造工程。

　　6.投入7000万元，建设或购买1000户廉租房。

 7.投入960万元，扩大廉租房租房户补贴范围，达到1万户，保障对象由人均建筑面积8平方米以下提高到10平方米以下。

　　十是抓好直接利民惠民工程。把改善民生作为一切工作的出发点和落脚点，坚持就业是民生之本、创业是就业之源的理念，努力提高人民群众的收入水平。实施“五险”扩面，完善救助体系，提高社会保障水平。具体要办好15件实事。

　　1.投入3500万元，为公益性岗位从业人员落实岗位补贴和社保补贴政策。

 2.投入3600万元，继续落实灵活就业社保补贴政策。

 3.发放小额担保贷款1000万元，发放范围扩大到大中专毕业生、转业复员军人、外出务工返乡、登记失业人员等所有创业人员，贷款额度由2万元提高到5万元。

 4.投入1100万元，免费开展城乡失业人员就业创业培训。

 5.投入4800万元，启动城镇居民医疗保险工作，参保率达到50％。

 6.投入423万元，将失业保险由每人每月196元提高到260元。

 7.启动新型农村养老保险试点，参保率达到15％。

 8.投入1200万元，使城市低保补助标准达到全省中上游水平。

 9.提高社区工作者生活补贴，现职社区主任、副主任每月提高100元，离岗的按工作年限给予生活补贴。

 10.全部兑现机关事业单位人员调资和公务员津贴补贴。

 11.投入300万元，建设为老服务综合楼。

 12.投入200万元，建设妇儿活动中心。

 13.投入450万元，开工建设老年活动中心。

 14.投入400万元，开工建设未成年流浪者保护中心。

 15.投入500万元，完成20个村整村扶贫开发任务。

　　（五）切实改善发展条件，为保增长保民生提供良好的环境保障

　　优良的环境是干事创业的基本条件，尤其是在今年世界大局动荡不安、国家经济形势严峻、我市加快发展面临诸多压力和矛盾的情况下，更需要全市人民共同营造团结、和谐、安定的发展氛围，使我们把主要精力投放到保增长、保民生上来。

　　一要大力改善安全生产环境。坚持“安全第一、预防为主、综合治理”的方针，巩固“百日安全会战”成果，开展“安全生产年”活动，切实扭转被动局面。继续实施以煤矿企业、人员密集场所、公路交通、建筑施工等为重点的专项整治行动和隐患排查治理。深刻吸取两起事故的教训，进一步落实区级政府和煤矿企业的两个主体责任，全面推进标准化和本质安全型煤矿建设。加强市场监管，深入开展以食品、药品安全为重点的专项整治，坚决防止假冒伪劣产品损害生产生活。

　　二要大力改善社会治安环境。把社会治安工作摆在更加突出的位置，强化综合治理，加强依法治市，实现打击、防范、教育有机结合。加强公安队伍建设，着力构建社会、社区、农村等八大防控体系，严厉打击各种犯罪，决不让治安问题影响正常生活和发展大局，切实增强人民群众和外来投资者的安全感，努力把鹤岗建成平安城市。

　　三要大力改善信访稳定环境。把解决群众实际问题作为首要任务，进一步强化信访责任制和主要领导接访制度，尽力解决信访案件。坚持依法治访，引导群众依法、理性、文明地表达诉求，果断处置违反《信访条例》和《治安管理处罚法》的行为，确保社会和谐稳定。依法妥善处理“民间借贷和集资”问题，尽一切努力降低群众损失，帮助合法经营的企业解决实际困难，坚决防止损害大局的事情发生。

　　四要大力改善经济发展环境。努力改善招商引资环境，坚决纠正行业不正之风，加强执法、执纪、执收部门的管理，强化重商安商的氛围。规范招商引资行为，杜绝各区之间互挖税源、互抢项目，防止税源流失和税收级次混库。依法整顿金融秩序，规范中介组织，加强诚信建设，创建金融生态区。

　　五要大力改善外部发展环境。扩大国际合作，加强与俄犹太州、日本鹤冈等国际友好州县的联系，拓展发展空间；加强国内交流，与北京、上海等友好市区和发达地区建立更多的合作关系；密切与国家和省直部门的联系，全力争取支持；充分发挥与鹤岗有渊源的成功人士的作用，为招商引资、项目建设提供更多支持。

　　（六）切实加强自身建设，为保增长保民生提供坚强的组织保障

　　复杂的形势、艰巨的任务，对政府工作提出了新的更高的要求。各级政府及其部门要抓住开展学习实践科学发展观活动的重要契机，以效能建设为载体，以班子建设为重点，以文化建设为基础，全面加强自身建设，努力使政府工作更加符合时代和人民群众的要求。

　　一要在解放思想、统一思想上下功夫。越是在关键时期，越是面对困难，越需要解放思想、统一思想。解放思想，就是要彻底摆脱因循守旧、安于现状的观念，敢于突破、敢于创新，善于采取超常规的办法打开局面。统一思想，就是要把思想统一到科学发展观上来，统一到中央对形势的判断和部署上来，统一到鹤岗发展的大局上来，真正做到一心一意干事业、群策群力求发展。要全面提高综合素质，切实增强大局意识、危机意识、忧患意识，善于在经济动荡中学习经济，在金融危机中学习金融，在纷繁复杂的矛盾中解决实际问题。要正确对待发展中的问题，妥善解决前进中的矛盾，进一步增强政治责任感，切实做到不因踌躇而失机遇，不因私利而害大局，不因时艰而误长远。

　　二要在依法行政、政务公开上下功夫。进一步强化法治观念，严格按照法定权限和程序行使权力、履行职责，做到依法决策、依法办事。进一步扩大政务公开，市政府领导分工、政府部门职责、重大决策、重点工作等信息，一律在主要媒体和政府网站公布，同时在振兴广场和市政府办公楼设置政务公开电子显示屏，随时公布有关信息。强化“12345”市长热线功能，确保群众诉求得到及时处置。要扩大公共财政覆盖范围，逐步将财政转向“民生财政”，使预算成为“民生预算”；要切实树立过紧日子的思想，坚决压缩一般性支出，把有限的财力重点投放到保民生、保发展、保稳定上来；要严格执行预算制度，控制各种临时支出、提前支出。要主动自觉地接受人大的法律监督和政协的民主监督，充分发挥群众监督和舆论监督的作用，不断提高政府工作的透明度和公信度。

　　三要在民主决策、强化服务上下功夫。健全科学民主的议事决策规则，组建市政府项目建设顾问组、城市规划专家组，实施集体领导、专家指导、群众参与的决策机制，严格实行重大事项听证、论证制度，推进决策的科学化、民主化。要强化激励机制，设立政府工作创新奖、人民建议奖和市民荣誉奖，充分发挥全市人民加快发展的积极性和创造性。要加强公共服务，进一步深化行政体制改革和政府机构改革，突出社会管理和公共服务职责。重点加强“五个中心”建设，即：完善人民办事中心，改善办事办公条件，提高服务质量；完善政府采购中心，规范采购行为，建立评价和监督体系；筹建公共资源交易中心，工程招投标、土地招拍挂等一律公开公示，接受社会监督；建设信访接待中心，实行市领导定期接访和部门派驻专人接访制度，为上访群众提供“一站式”服务；建设突发事件应急处置中心，形成快速反应、有效处置的工作机制。

　　四要在提升效能、改进作风上下功夫。深入开展效能建设，重点开展“五治”活动。即：通过治庸，解决能力低下、无所作为的问题；通过治懒，解决效率低下、无所事事的问题；通过治乱，解决不讲程序、不守规矩的问题；通过治虚，解决不讲真话、不干实事的问题；通过治腐，解决不干不净、刁难勒卡的问题。全面加强作风建设，强化“严、细、深、实、勤、俭、廉”七种作风，切实提高办事效率、工作质量和干事能力，努力建设服务政府、法制政府、责任政府和创新政府。

　　各位代表、各位同志，我们深知今年形势严峻，任务艰巨，也深知人民群众的要求和期望。我们坚信，有市委的正确领导，有全市人民的大力支持，我们一定能够战胜各种困难，冲破各种难关，全面完成既定的任务目标，以优异的成绩向全市人民交上一份满意的答卷！
